# Modified Vaccinia virus Ankara but not vaccinia virus induces chemokine expression in cells of the monocyte/macrophage lineage

**DOI:** 10.1186/s12985-015-0252-1

**Published:** 2015-02-12

**Authors:** Michael H Lehmann, Philip JR Price, Christine Brandmüller, Gerd Sutter

**Affiliations:** Institute for Infectious Diseases and Zoonoses, Ludwig-Maximilians-Universität München, Munich, Germany; German Centre for Infection Research (DZIF), Partner site, Munich, Germany

**Keywords:** Alveolar macrophage, CCR1, Chemotaxis, CXCR2, MH-S, MPRO, MVA, Neutrophil, THP-1, U937

## Abstract

**Background:**

The orthopoxvirus strain Modified Vaccinia virus Ankara (MVA) rapidly induces innate immune responses. Previously, we demonstrated that CCL2 and CCR1 are important players in MVA induced recruitment of leukocytes to the lung. Alveolar macrophages are sentinel cells in the lung, which are likely amongst the first cells of the immune system to encounter and respond to virus during respiratory infection. Therefore we examined the potential of the murine alveolar macrophage MH-S cell line as a model to study chemokine expression during infection with MVA and vaccinia virus (VACV) strain Western Reserve (WR).

**Findings:**

MVA but not VACV infected MH-S cells increased the expression of the CXCR2 acting chemokine CXCL2. MH-S cells constitutively produced CCL2 and CCR1 acting chemokines CCL3, CCL5 and CCL9. Consequently, supernatants of mock treated and virus infected MH-S cells induced chemotaxis of murine promyelocyte MPRO cells and human monocytic THP-1 cells at the same level. However, supernatants of MVA infected MH-S cells significantly increased chemotaxis of the CCR2 deficient human monocytic cell line U-937. Chemotaxis of all three cell types was inhibited by J 113863, a CCR1 antagonist. Additionally, we show that MVA but not VACV WR infection of THP-1 cells induces expression of C-C motif and C-X-C motif chemokines and generates a chemotactic activity for monocytes, which was J 113863 sensitive.

**Conclusions:**

These results extend our previous findings, demonstrating that MVA but not VACV WR induces chemokine production in alveolar macrophages and monocytes, which can induce recruitment of monocytes in a CCR1 dependent manner.

**Electronic supplementary material:**

The online version of this article (doi:10.1186/s12985-015-0252-1) contains supplementary material, which is available to authorized users.

## Findings

Highly attenuated, replication deficient vaccinia strains such as Modified Vaccinia virus Ankara (MVA) are increasingly used as viral vectors for the development of new vaccines. Intranasal inoculation is an attractive route of vaccination for delivery of MVA to mucosal tissue in the respiratory tract, which was found to be both safe and immunogenic in a phase I clinical trial [1]. A distinguishing feature of MVA is its ability to activate a robust innate immune response [2-4]. We recently showed that MVA triggers the production of chemokines in primary lung fibroblasts and bone marrow derived macrophages (BMDM) which induce chemotaxis of neutrophils [5].

Alveolar macrophages (AM) are permanent residents of the lung which are capable of exerting both pro- and anti-inflammatory immune responses. Due to their presence on the luminal surface of the respiratory system AM are likely amongst the first cells to encounter virus after respiratory infection. Indeed, it has been shown that AM help to control VACV infection, and their depletion exacerbates the host inflammatory response to viral infection, which is probably due to the increased viral burden [6]. However little is known about the early immune responses that is elicited in AM upon encounter with VACV or highly attenuated strains such as MVA. Activation of AM is tightly regulated by the local microenvironment, cell-cell interactions and soluble mediators that inhibit activation of pro-inflammatory signalling pathways, and removal of these signals leads to spontaneous activation of AM [7]. Consequently, our attempts to investigate cytokine expression in AM *ex-vivo* were largely unsuccessful due primarily to strong cytokine production in non-infected cells and rapid cell death of infected cells after isolation by bronchoalveolar lavage (data not shown). Therefore we tested the immortalized murine alveolar macrophage MH-S cell line [8] as a model system for MVA induced chemokine expression.

**Figure 1:**
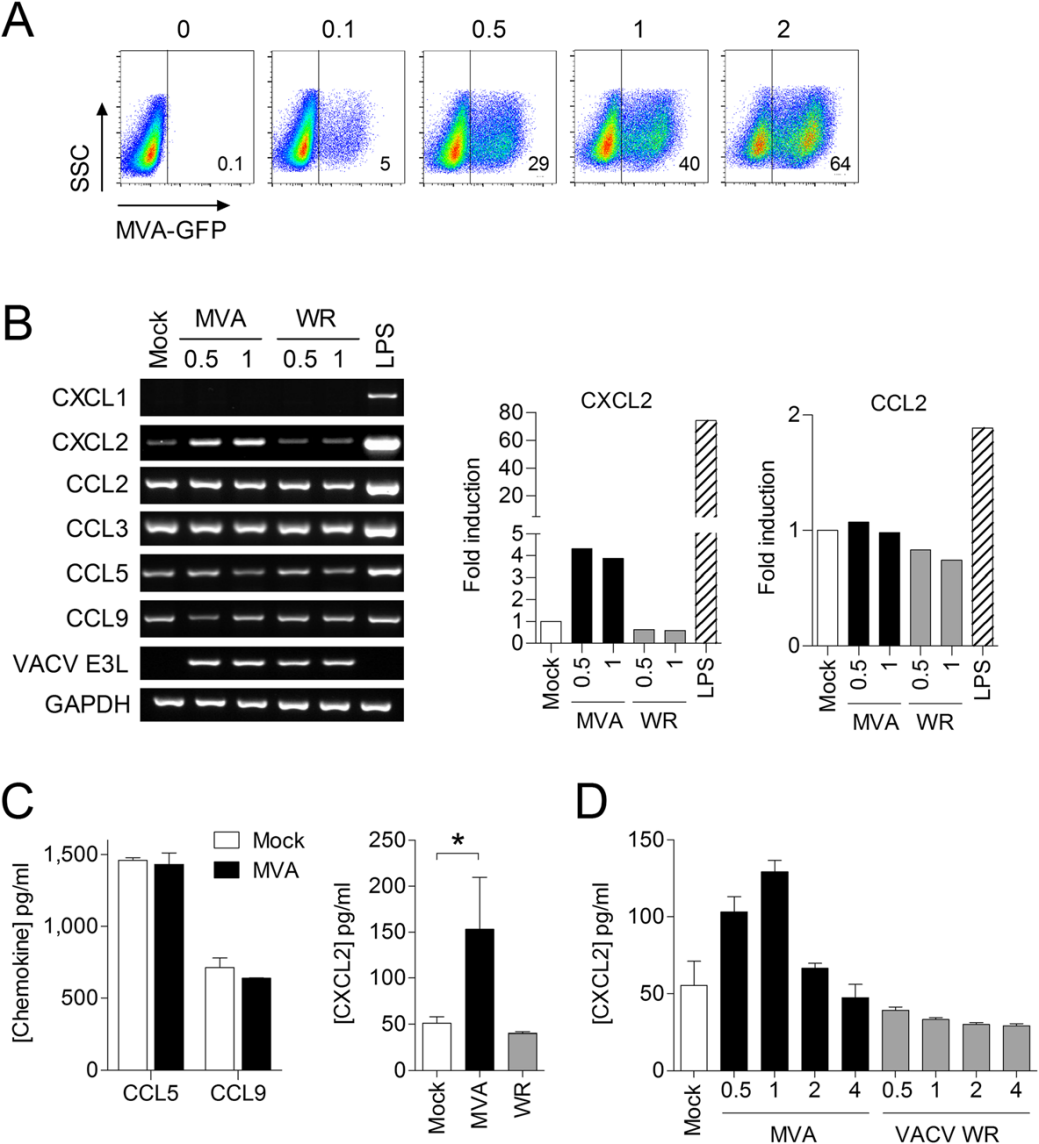
**Murine alveolar macrophage MH-S cells produce neutrophil-attracting chemokines constitutively and after infection with MVA. A)** MH-S cells were infected with MVA-GFP at the indicated MOI and infectivity was measured 16 h p.i. by flow cytometry. **B)** MH-S cells were infected with MVA or VACV WR at an MOI of 0.5 and 1, or challenged with LPS (1 μg/ml). Total RNA was isolated 8 h p.i. and investigated subsequently by specific RT-PCR as indicated. Fold induction of CXCL2 and CCL2 were calculated based on the intensity of PCR products using Image Lab 5.0 (Bio-Rad). Data from mock-treated cells were set to 1. **C)** Cell culture supernatants of MVA and VACV WR infected MH-S cells (1 MOI) were harvested 8 h p.i. and **D)** 16 h p.i., and chemokine concentrations determined by specific ELISA. Data are means ± SD and are representative of at least two independent experiments, *, P < 0.05; ANOVA with Bonferroni post-hoc test.

To determine whether MH-S cells are permissive to vaccinia virus infection and gene expression, cells were infected with MVA expressing the green fluorescent protein (GFP) under control of the P7.5 early/late promoter [9], and analysed using a MACSQuant VYB flow cytometer (Miltenyi Biotec). This showed that MH-S cells are readily infected with MVA and are permissive to viral gene expression (Figure 1A). Analysis by RT-PCR and ELISA showed that the CXCR2 ligand CXCL2 was induced by MVA but not by VACV WR in MH-S cells confirming our previous finding in primary murine lung fibroblasts [5]. Increased mRNA and protein levels of CXCL2 were detected at 8 h p.i., and the amount of CXCL2 produced depended on the MOI applied (Figure 1B, C and D). CXCL1 was only induced by LPS in MH-S cells, whereas CXCL5/6 (GCP-2/LIX), another neutrophils attracting chemokine [10], was not. Functionality of the GCP-2 specific PCR was demonstrated using RNA from murine alveolar epithelial MLE-12 cells treated with LPS or with supernatant from LPS challenged MH-S cells (Additional file 1).

Recently, we showed that CCR1 plays an important role in the recruitment of CD11b^+^Ly6C^hi^ inflammatory monocytes into the lung [5]. Monocytes express the chemokine receptor CCR2 [11], and CCL2, a ligand for this receptor, has been shown to be important for MVA induced monocyte migration *in vitro* and *in vivo* [12]. However**,** monocytes are a heterogeneous population, and some subsets do not express CCR2 [13]. Moreover, CCR1 expression is up-regulated on differentiating monocytes, leading to increased sensitivity to CCR1 ligands whilst simultaneously decreasing sensitivity to CCR2 ligands [14]. Consequently, we asked whether CCR1 also plays a role in mediating MVA triggered monocyte chemotaxis.

**Figure 2:**
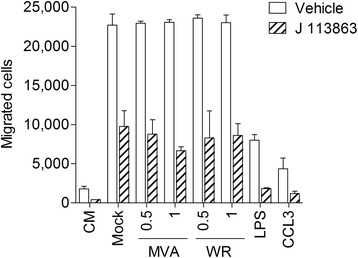
**Supernatants from MH-S cells increase chemotaxis of murine promyelocyte MPRO cells in a CCR1 dependent manner.** MPRO cells were tested for chemotaxis towards control medium (CM), human CCL3 (20 ng/ml) and supernatants from MH-S cells (16 h) challenged with LPS (1 μg/ml) or infected with MVA or VACV WR at an MOI as indicated using a 96-well Multi-Screen-MIC plate with a 5 μm pore-sized filter (Millipore). 75.000 cells were placed in the upper part and cells migrated into the lower part were counted after 3 h. Where indicated MPRO cells were pre-incubated with 20 nM of the CCR1 antagonist J 113863 for 5 min prior to running the assay. Data are means ± SD (n = 3).

Unfortunately, the CCR1 ligands CCL3, CCL5 and CCL9, as well as the CCR2 ligand CCL2, were constitutively expressed in MH-S cells, and the levels were not increased by MVA or VACV WR infection (Figure 1B and C). Consequently, chemotaxis of murine promyelocyte MPRO cells was increased by supernatants from mock and virus infected MH-S cells to a similar level and decreased when MPRO cells were pre-incubated with the CCR1 antagonist J 113863 (Figure 2). Supernatants from LPS challenged MH-S cells induced less chemotaxis as compared to supernatants from mock infected cells. A possible explanation is that LPS-induced interleukin-10 in MH-S cells may have decreased the constitutive protein production of the C-C motif chemokines in a negative autocrine feedback loop [15,16].

**Figure 3:**
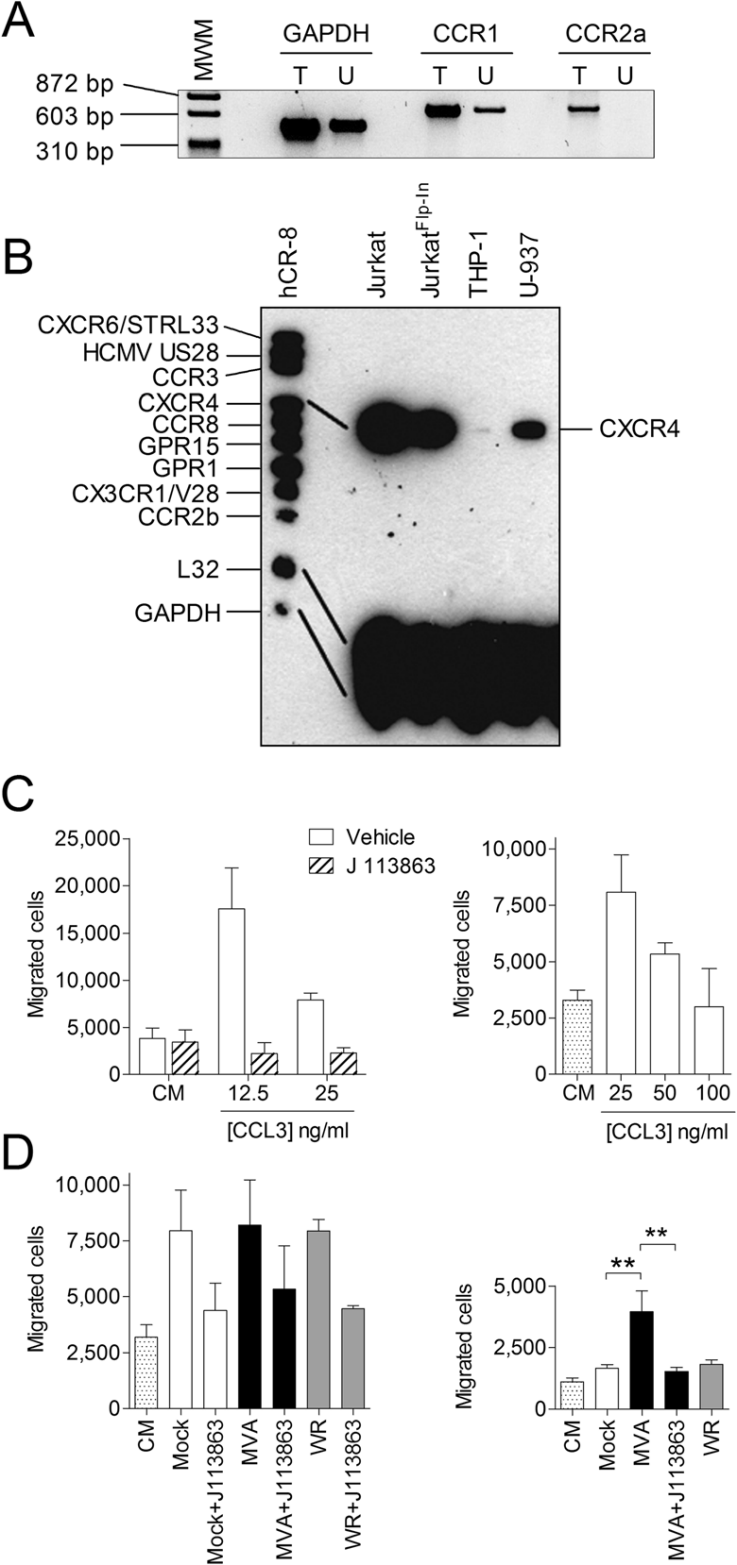
**Supernatants from MVA but not VACV WR infected MH-S cells increase chemotaxis of U-937 cells in a CCR1 dependent manner. A)** Total RNA from THP-1 cells (T) and U-937 cells (U) were isolated and used for GAPDH, CCR1 and CCR2 transcript variant A specific RT-PCR. **B)** Total RNA was isolated from cells as indicated and a multiprobe ribonuclease protection assay using hCR-8 (BD Biosciences) as probe was performed as previously described [12]. **C)** Verification of J 113863 to block CCR1 specific chemotaxis of THP-1 cells. Human CCL3 at concentrations as indicated was added to the lower part of a 96-well Multi-Screen-MIC plate with an 8 μm pore-sized filter (Millipore). 75.000 cells were placed in the upper part and cells migrated into the lower part were counted after 90 min. Data are means ± SD (n = 3). **D)** Human monocytic THP-1 cells (left panel) and U-937 cells (right panel) were tested for chemotaxis towards control medium (CM) and supernatants from mock, MVA or VACV WR infected MH-S cells (1 MOI, 16 h) as described in C). Where indicated, cells were pre-incubated with 5 nM of the CCR1 antagonist J 113863 for 5 min prior to running the assay. THP-1 cells and U-937 cells were allowed to migrate for 90 min and 30 min, respectively. Data are means ± SD (n = 3 for THP-1 cells; n = 6 for U-937 cells) and are representative of at least two independent experiments, **, P < 0.01; ANOVA with Bonferroni post-hoc test.

To avoid the masking effect of CCL2 when investigating MVA for its potential to induce a CCR1 dependent chemotactic activity in cells of the monocyte/macrophage lineage we took advantage of the human monocytic cell line U-937, which, under our cell culture conditions, did not express CCR2 in contrast to human monocytic THP-1 cells (Figure 3A and B) [17]. Firstly, we tested whether supernatants from MH-S cells are also capable of inducing chemotaxis of THP-1 cells. This was possible since human chemokine receptors CCR1 and CCR2 can also be activated by the relevant murine ligands [18-20]. As with MPRO cells, supernatants from mock and virus infected MH-S cells increased chemotaxis of THP-1 at a similar level, which was decreased when THP-1 cells were pre-incubated with J 113863 (Figure 3D, left panel). Supernatants from MVA infected MH-S cells increased the chemotaxis of U-937 cells compared to supernatants from mock infected and VACV WR infected MH-S cells. Importantly, this chemotaxis of U-937 cells was significantly reduced when U-937 cells were pre-incubated with J 113863 (Figure 3D, right panel). Of note, J 113863 also acts on human CCR3 but this receptor was not expressed in either cell line (Figure 3B). Interestingly, despite the constitutive expression of CCR1 ligands by MH-S cells, levels of chemotaxis of U-937 towards culture supernatants of mock-infected cells were only slightly increased as compared to medium control (Figure 3D). This potentially indicates that other mechanisms induced by MVA may be required for optimal induction of cell migration.

In a recent study, we showed that complement component C5 is required for respiratory recruitment of neutrophils during MVA infection [21]. Surprisingly, complement component C3 was not necessary, which suggests the activation of an unknown proteolytic process by MVA. Indeed, CCL3 is N-terminally processed by CD26/dipeptidyl peptidase IV converting it to a very potent CCR1 ligand [22]. Taken together, in future studies it would be interesting to investigate whether an MVA activated protease plays a role in MVA triggered recruitment of monocytes and neutrophils.

**Figure 4:**
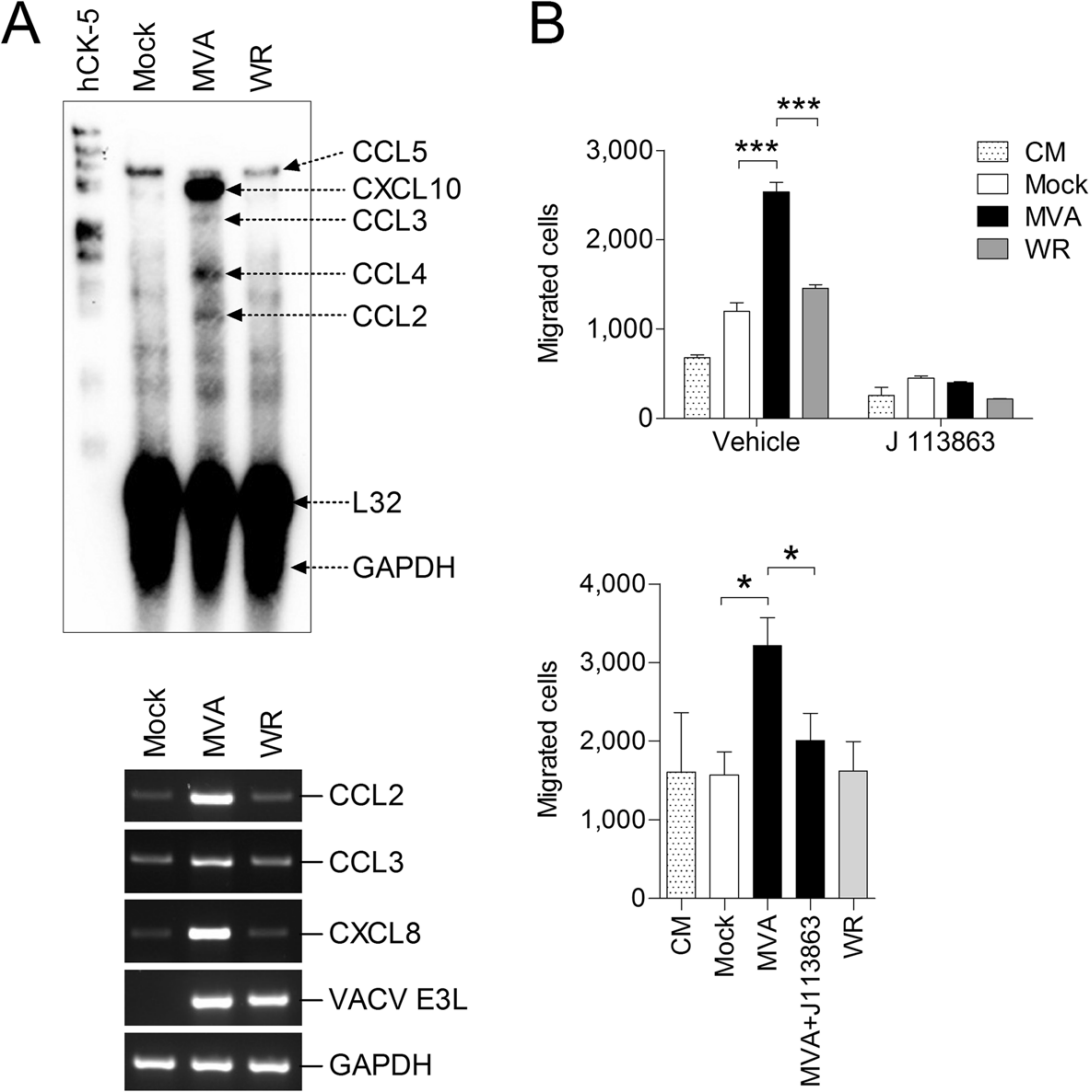
**MVA, but not VACV WR, induces chemokine production in THP-1 cells and increases chemotaxis of monocytes in a CCR1 dependent manner. A)** Total RNA from MVA or VACV WR infected THP-1 cells were isolated 6 hours p.i. and investigated subsequently by multiprobe ribonuclease protection assay using hCK-5 as probe (upper panel) and specific RT-PCR as indicated (lower panel). **B)** Chemotaxis of THP-1 cells (upper panel) and U-937 cells (lower panel) toward control medium (CM) and culture supernatants from THP-1 cells infected with MVA or VACV WR at 1 MOI for 16 hours. Where indicated, cells were treated with 5 nM of CCR1 antagonist J 113863 or the equivalent amount of solvent (all other samples) before running the assay as described in Figure 3. Data are means ± SD (n = 4). *, P < 0.05. ***, P < 0.001.

Previously we showed that MVA, but not VACV strain Elstree, induces chemokine expression including CCR1 acting chemokines in THP-1 cells [12]. Here we confirm our previous finding using multiprobe ribonuclease protection assay (RPA) and RT-PCR. Additionally we demonstrated that VACV WR does not induce chemokine expression in THP-1 cells (Figure 4A) and that supernatants from MVA but not VACV WR infected human monocytic THP-1 cells significantly increase chemotaxis of both naïve THP-1 cells and U-937 cells as compared to supernatants from mock infected cells (Figure 4B). As a side note, treatment of cells with the CCR1 antagonist J 113863 reduced background levels of THP-1 cell chemotaxis towards supernatants from mock infected cells and medium controls. This indicates that there was a CCR1 acting ligand present not only in supernatants of mock infected cells but also in the cell culture medium used. Indeed, constitutive expression of CCL5 in THP-1 cells was detected by multiprobe RPA, which may contribute to that effect (Figure 4A). Additionally, a bovine CCL3 isoform capable of inducing THP-1 chemotaxis in a CCR1 dependent manner has been detected in calf serum [23], and the cell culture medium used here for chemotaxis assays regularly contained 0.5% fetal calf serum.

In summary, we demonstrated that MH-S cells can be used as model system to study MVA infection in alveolar macrophages but due to the high constitutive production of C-C motif chemokines only limited conclusions can be drawn from functional studies where the involvement of these proteins is relevant. Nevertheless, the results presented here confirm our recent findings in primary murine lung fibroblasts and bone marrow derived macrophages showing additionally that MVA, but not VACV WR, induces chemokine expression in cells of the monocyte/macrophage lineage that is capable of inducing chemotaxis of neutrophils and monocytes.

## Additional file

Additional file 1:
**Verification of RT-PCR for murine CXCL5/GCP-2/LIX.** As indicated, MLE-12 cells were challenged with LPS (1 μg/ml), with supernatants from MH-S cells (snMH-S) or with supernatants from LPS-treated MH-S cells (snMH-S^LPS^) for six hours and subsequently lysed for total RNA isolation. RNA was converted in cDNA and amplified using forward primer 5'-catttctgttgctgttcacg-3' and reverse primer 5'-atacatattccggagacaatgc-3'. Primers were designed using NCBI Reference Sequence: NM_009141.3; Mus musculus chemokine (C-X-C motif) ligand 5 (Cxcl5), mRNA. The CXCL5/GCP-2/LIX PCR product corresponded with the expected size of 617 bp. RT-PCR for murine GAPDH was performed as described (5). PCR without cDNA served as negative control. Lambda DNA Hind III/phiX174 DNA Hae III was used as molecular weight marker (MWM).
