# Supplementary figures and images for: Modified Vaccinia virus Ankara but not vaccinia virus induces chemokine expression in cells of the monocyte/macrophage lineage
Source: Virol J. 2015 Feb 12;12:21. doi: 10.1186/s12985-015-0252-1 (PMC4349667; doi:10.1186/s12985-015-0252-1)

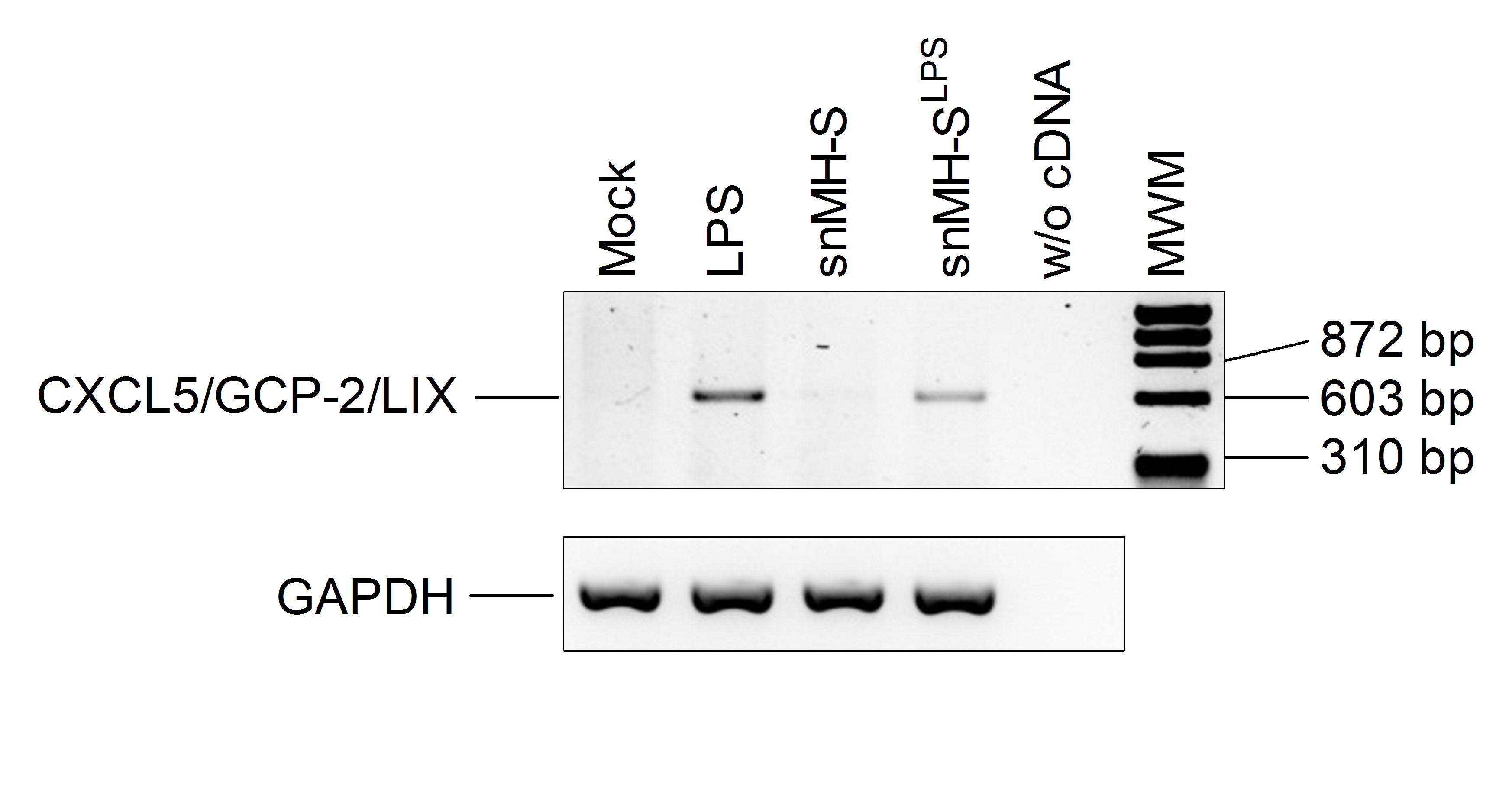

Supplement: Additional file 1: — Verification of RT-PCR for murine CXCL5/GCP-2/LIX. As indicated, MLE-12 cells were challenged with LPS (1 μg/ml), with supernatants from MH-S cells (snMH-S) or with supernatants from LPS-treated MH-S cells (snMH-SLPS) for six hours and subsequently lysed for total RNA isolation. RNA was converted in cDNA and amplified using forward primer 5'-catttctgttgctgttcacg-3' and reverse primer 5'-atacatattccggagacaatgc-3'. Primers were designed using NCBI Reference Sequence: NM_009141.3; Mus musculus chemokine (C-X-C motif) ligand 5 (Cxcl5), mRNA. The CXCL5/GCP-2/LIX PCR product corresponded with the expected size of 617 bp. RT-PCR for murine GAPDH was performed as described (5). PCR without cDNA served as negative control. Lambda DNA Hind III/phiX174 DNA Hae III was used as molecular weight marker (MWM). [file 12985_2015_252_MOESM1_ESM.tiff]
